# Surgical Aspects of Corpus Callosotomy

**DOI:** 10.3390/brainsci11121608

**Published:** 2021-12-05

**Authors:** Takehiro Uda, Noritsugu Kunihiro, Ryoko Umaba, Saya Koh, Toshiyuki Kawashima, Shohei Ikeda, Kotaro Ishimoto, Takeo Goto

**Affiliations:** 1 Department of Neurosurgery, Graduate School of Medicine, Osaka City University, Osaka 545-8585, Japan; onaka0402@gmail.com (S.K.); toshiyuki1986.331.24ser@gmail.com (T.K.); ikdx81@gmail.com (S.I.); noukotaro@gmail.com (K.I.); gotot@med.osaka-cu.ac.jp (T.G.); 2 Department of Pediatric Neurosurgery, Osaka City General Hospital, Osaka 534-0021, Japan; nori9216@med.osaka-cu.ac.jp (N.K.); ryocoumaba@gmail.com (R.U.)

**Keywords:** corpus callosotomy, surgical technique, disconnection syndrome, complication, endoscopy

## Abstract

Corpus callosotomy (CC) is one of the options in epilepsy surgeries to palliate patient seizures, and is typically applied for drop attacks. The mechanisms of seizure palliation involve disrupting the propagation of epileptic activity to the contralateral side of the brain. This review article focuses on the surgical aspects of CC. As a variations of CC, anterior two-thirds, posterior one-third, and total callosotomy are described with intraoperative photographs. As less-invasive surgical variations, recent progress in endoscopic CC, and CC without craniotomy, is described. CC remains acceptable under the low prevalence of complications, and surgeons should make the maximum effort to minimize the complication rate.

## 1. Introduction

The corpus callosum is the largest bundle of commissural fibers connecting the two hemispheres of the brain. Most epileptic discharges generated in one hemisphere that propagate contralaterally pass through the corpus callosum. Severing the corpus callosum can thus disrupt the contralateral propagation of epileptic activity, and seizures may thus be palliated. This is the consensus explanation of the mechanism underlying the efficacy of corpus callosotomy (CC). The present review article provides details of the surgical aspects of CC, reviewing the previous literature.

## 2. Indications for CC

When patients still experience intractable seizures despite taking multiple anti-seizure drugs, surgical interventions for epilepsy can be considered. The best target seizure for CC is drop attack, a kind of seizure semiology caused by atonic seizures, tonic seizures, epileptic spasms, and myoclonic seizures, that occasionally results in trauma to the forehead and face of the patient. For bedridden patients, while these seizures do not cause drop attacks per se, CC remains a viable option, depending on the severity of the seizures. CC can also be applied for tonic seizures, secondary generalization of focal seizures, absence seizures, epileptic spasms, and myoclonic seizures [1,2,3]. The typical interictal electroencephalography findings of patients suggested for CC are bilateral synchronized or multifocal independent epileptic spikes.

## 3. Disconnection Syndrome and Extent of Corpus Callosum Disconnection

Disconnection syndrome is the main concern after CC [4], and can be separated into acute and chronic disconnection syndromes. Particularly in acute disconnection syndrome, patients suffer from severe decreases in spontaneous speech, paresis of the nondominant leg, and incontinence. The severity of symptoms varies in each, but patients might experience severe loss of activity that necessitates tube feeding or intravenous total parenteral nutrition. Another possible problem is severe dysphagia, which can lead to aspiration pneumonia. Such symptoms gradually improve over a period of weeks to months [5]. In chronic disconnection syndrome, patients can suffer from various symptoms such as alien hand syndrome, dichotic listening suppression, tactile dysnomia, hemispatial neglect, nondominant hand agraphia, and tachistoscopic visual suppression. The severity of the symptoms, again, varies in each, but can lead to disability in more elderly patients.

Total CC (tCC) is superior to anterior two-thirds callosotomy (aCC) in terms of seizure outcomes [3,6,7]. However, aCC preserves the splenium of the corpus callosum, and is therefore applied in older patients to avoid disconnection syndrome. Criteria to apply aCC instead of tCC have yet to be standardized. In our institution, patients over 15 years old undergo aCC and patients under 10 years old undergo tCC. In patients between 10 to 15 years old, the extent of CC is determined depending on the activities of daily living, cognitive functions, and findings from electroencephalography. In cases with insufficient seizure control after aCC, additional posterior one-third callosotomy (pCC) is applied at least three months later. In limited cases, pCC is applied alone. However, in recent reports, pCC alone has offered sufficient seizure control rates for drop attacks [8,9].

## 4. Surgery

### 4.1. Microsurgical tCC and aCC

#### 4.1.1. Position, Skin Incision and Craniotomy

**Figure 1:**
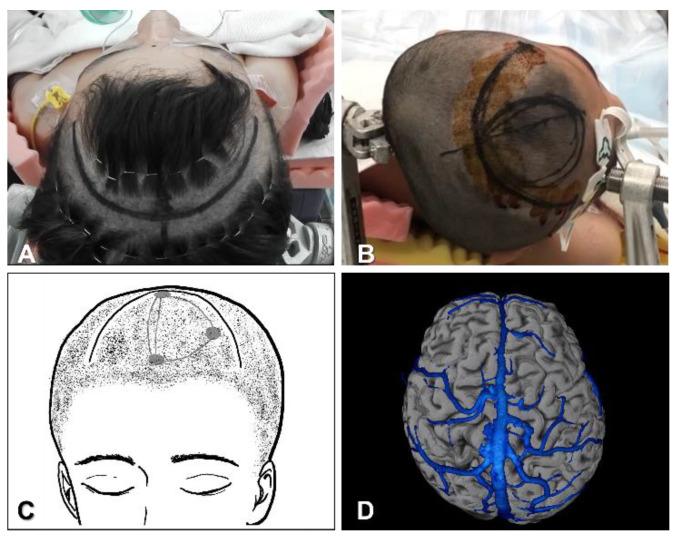
Patient position, skin incision and craniotomy in anterior or total corpus callosotomy. (**A**): Supine position without neck rotation. Black line indicates the skin incision. (**B**): Lateral position with entry side down. (**C)**: Drawing of the incision line and craniotomy. (**D**): Venography with contrast-enhanced computed tomography to determine the side of entry.

Most surgeons set the patient supine without neck rotation (Figure 1A), while others prefer to set the patient in a lateral position with the entry side down, to allow dissection of the interhemispheric fissure with the aid of the effects of gravity on the brain (Figure 1B). The skin is incised coronally. The diameter of the craniotomy is approximately 6 cm (Figure 1C).

The coronal suture represents a posterior margin for the craniotomy. Frontal craniotomy is performed 1 cm beyond the midline. Craniotomies around the central sulcus should be avoided in most cases, because the largest bridging vein to the superior sagittal sinus (SSS) runs near the central sulcus, as a so-called vein of Trolard. The side of entry to the interhemispheric fissure also depends on the bridging veins. Venography with contrast-enhanced computed tomography or magnetic resonance imaging is useful to identify bridging veins to the SSS (Figure 1D).

#### 4.1.2. Interhemispheric Fissure Dissection

**Figure 2:**
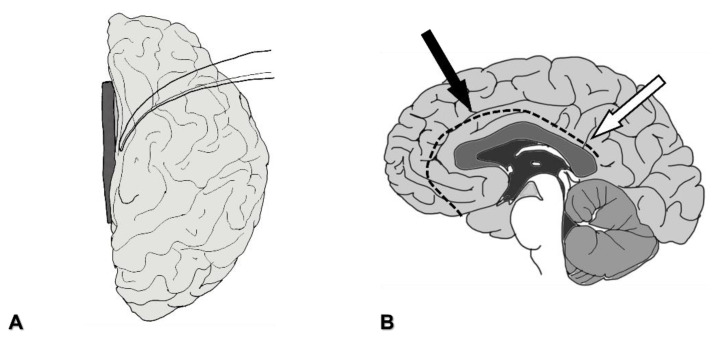
The direction of the entry to the corpus callosum. (**A**): After dural incision, the frontal lobe is retracted laterally with the spatula. (**B**): Black arrow indicates the direction toward the corpus callosum in anterior corpus callosotomy and total corpus callosotomy. White arrow indicates the direction toward the corpus callosum in posterior corpus callosotomy. Dotted line indicates the inferior edge of the falx cerebri.

**Figure 3:**
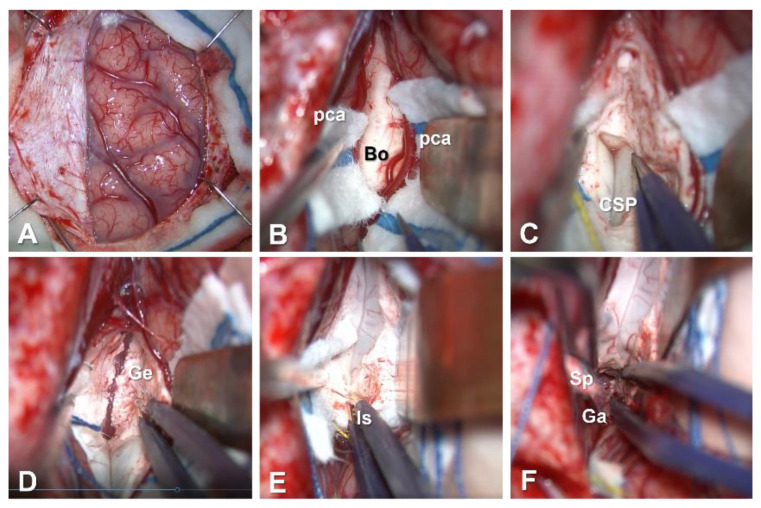
Intraoperative photographs in microsurgical total corpus callosotomy. (**A**): Brain surface after dural incision. (**B**): After opening the interhemispheric fissure, the body of the corpus callosum is seen between bilateral pericallosal arteries. (**C**): The cavum of the septum pellucidum is an anatomical landmark for severing the corpus callosum. (**D**): Callosal section of the genu and rostrum of the corpus callosum. (**E**): Isthmus of the corpus callosum. (**F**): Sectioning of the splenium of the corpus callosum. The vein of Galen is seen through the arachnoid membrane. (Bo: body of the corpus callosum; CSP: cavum of the septum pellucidum; Ga: vein of Galen; Ge: genu of the corpus callosum; Is: isthmus of the corpus callosum; pca: pericallosal artery; Sp: splenium of the corpus callosum.)

After the dural incision, the frontal lobe is retracted laterally (Figure 2A and Figure 3A). Large bridging veins from the frontal lobe to the SSS should be preserved as much as possible. On the medial side of the superior frontal gyrus, the falx cerebri separates the hemispheres. This structure ends at the level of the cingulate gyrus, and both hemispheres are tightly connected beyond the inferior edge of the falx cerebri (Figure 2B). To approach the corpus callosum without damaging the cerebral parenchyma, the interhemispheric fissure needs to be dissected meticulously. A meticulous sharp dissecting technique is necessary to prevent damage to the pia mater. Draining cerebrospinal fluid (CSF) from the arachnoid space results in the brain becoming slack. Repeated changes in the location of the spatula should be considered for effective retraction. Callosomarginal arteries, and then pericallosal arteries, are identified in the interhemispheric fissure. Between bilateral pericallosal arteries, the body of the corpus callosum is identified (Figure 3B).

#### 4.1.3. Severing the Corpus Callosum

**Figure 4:**
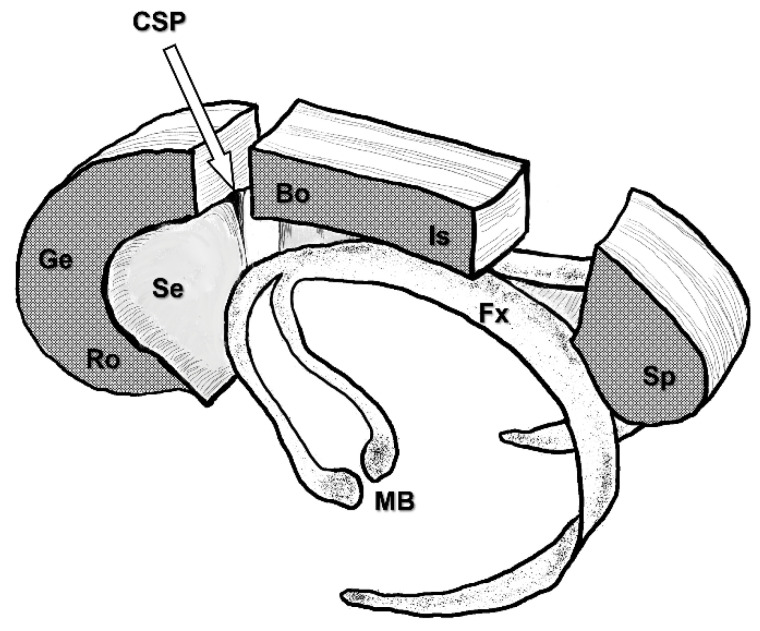
Three-dimensional relationships between the corpus callosum, fornix, and cavum of the septum pellucidum. (CSP: cavum of septum pellucidum; Bo: body of the corpus callosum; Fx: fornix; Ge: genu of the corpus callosum; Is: isthmus of the corpus callosum; MB: mammillary body; Ro: rostrum of the corpus callosum; Se: septum pellucidum; Sp: splenium of the corpus callosum.)

The corpus callosum, fornix, and cavum of the septum pellucidum (CSP) are depicted to demonstrate the three-dimensional relationships between them (Figure 4). Small vessels on the corpus callosum are coagulated and cut. Dissection of the corpus callosum is started with microscissors, forceps, a suction tube or an ultrasonic aspiration device. Meticulous dissection leads the surgeons to the CSP, which is an important landmark for achieving the disconnection of callosal fibers (Figure 3C). Even in cases where CSP appears invisible on preoperative coronal-section MRI, the CSP is identified in all cases. Along with CSP, dissection of the corpus callosum is continued anteriorly. The genu and rostrum of the corpus callosum are then dissected (Figure 3D). Thereafter, the optic axis of the microscope is changed posteriorly to dissect the posterior part of the corpus callosum (Figure 3E). In cases undergoing aCC, the transitional part between the body and isthmus of the corpus callosum represents the posterior end of the disconnection. Neuronavigation is useful to confirm this end. In cases undergoing tCC, the disconnection is continued posteriorly to the splenium of the corpus callosum. There, the upper wall of the cavum velum interpositum (CVI) is a landmark for achieving disconnection of callosal fibers, instead of the CSP. After disconnection of the splenium of the corpus callosum, a vein of Galen is confirmed through the arachnoid membrane (Figure 3F).

### 4.2. Microsurgical pCC

#### 4.2.1. Position, Skin Incision and Craniotomy

**Figure 5:**
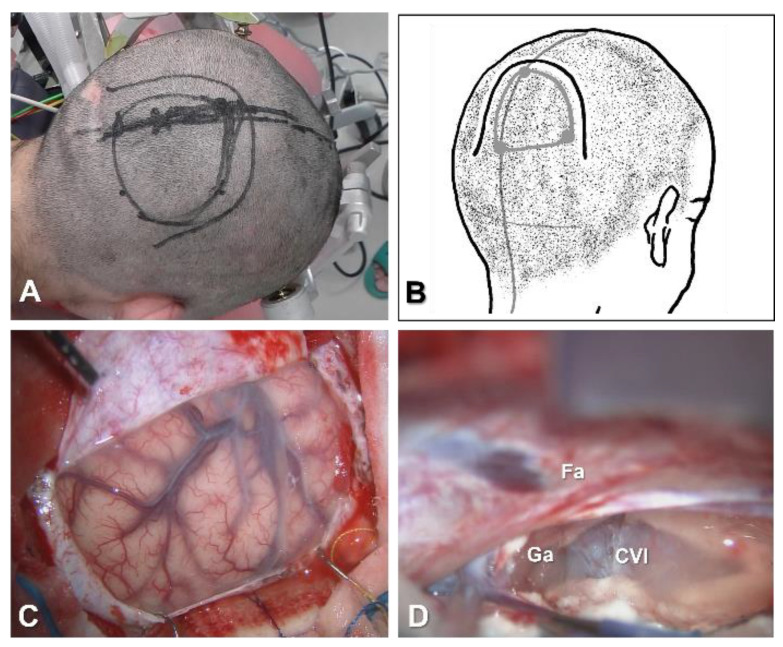
Patient positioning, skin incision, and craniotomy, and intraoperative photographs in microsurgical posterior corpus callosotomy. (**A**): In the semi-prone park-bench position with the entry side down. Black line indicates the skin incision and parieto-occipital craniotomy. (**B**): Drawing of the U-shaped skin incision and craniotomy. (**C**): Right parietal lobe after dural incision. (**D**): After posterior corpus callosotomy. The vein of Galen and upper wall of the cavum velum interpositum are seen. (CVI: cavum velum interpositum; Fa: falx cerebri; Ga: vein of Galen.)

In cases undergoing pCC after aCC, the anterior route to the posterior part of the corpus callosum is likely to show severe adhesions, so another, shorter, route is selected, with posterior craniotomy [10]. The patient is placed either in the semi-prone park-bench position, with the entry side down, or in the prone position (Figure 5A). A parieto-occipital craniotomy, 1 cm beyond the midline, is made around the lambdoid suture. The diameter of the craniotomy is approximately 5 cm. The skin is incised in a U-shape (Figure 5B).

#### 4.2.2. Interhemispheric Fissure Dissection

The parietal lobe is retracted laterally (Figure 5C). Neuronavigation is useful for following the shortest route to the posterior part of the corpus callosum. Draining CSF from the pericallosal cistern allows slackness in the brain tissue. Here, the inferior edge of the falx cerebri is close to the corpus callosum, and interhemispheric dissection is thus easier compared with the anterior part (Figure 2B). The posterior part of the corpus callosum is identified over the inferior edge end of the falx cerebri. Peripheral parts of the pericallosal arteries might be seen on the corpus callosum.

#### 4.2.3. Severing the Corpus Callosum

The posterior part of the corpus callosum is dissected using microscissors, forceps, a suction tube or an ultrasonic aspiration device. At the posterior end of the splenium, the vein of Galen is seen through the arachnoid membrane. At the inferior end, the upper wall of the CVI is seen (Figure 5D). At this point, the optic axis of the microscope is changed anteriorly to dissect the isthmus of the corpus callosum.

### 4.3. Endoscopic Corpus Callosotomy

The use of neuroendoscopy has recently become more common in the field of neurosurgery with the development of thin, high-resolution endoscopes. Compared with other neurosurgical fields, such as pituitary surgery, skull-base surgery, and intraventricular tumor surgery, the adoption of such applications is slower in the field of epilepsy surgery. However, along with the trend toward making surgeries less invasive, the use of endoscopes is becoming common in the field of epilepsy surgery.

**Figure 6:**
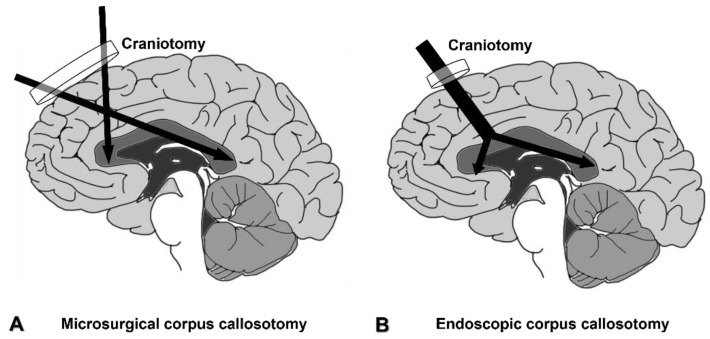
Comparison of craniotomy and surgical field between microsurgical (**A**) and endoscopic corpus callosotomy (**B**).

The endoscope is a useful surgical instrument for making the craniotomy smaller and reducing the length of the skin incision. Other possible advantages are lower complication rates, shorter surgical time, and shorter hospitalization. These advantages might justify the use of an endoscope; however, the number of reports regarding endoscopic corpus callosotomy is insufficient, and thus the direct comparison of those factors is difficult for now. Several cadaveric studies and case series regarding the use of endoscopes for corpus callosotomy have been reported [11,12,13,14]. An endoscope alone, or combined use of an endoscope and microscope, provides surgeons with a wider surgical field than microscopic surgery, particularly in deep areas (Figure 6).

**Figure 7:**
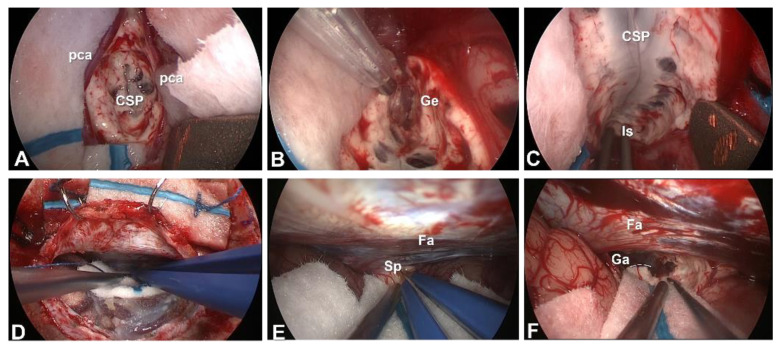
Intraoperative photographs from endoscopic anterior corpus callosotomy (**A**–**C**) and posterior corpus callosotomy (**D**–**F**). (**A**): After opening the interhemispheric fissure and severing the body of the corpus callosum, the cavum of the septum pellucidum is seen. (**B**): Callosal section of the genu and rostrum of the corpus callosum. (**C**): Isthmus of the corpus callosum. (**D**): After dural incision, showing retraction of the right parietal lobe. (**E**): Splenium of the corpus callosotomy at the inferior edge of the falx cerebri. (**F**): After sectioning of the splenium. The vein of Galen is seen through the arachnoid membrane. (CSP: cavum of the septum pellucidum; Fa: falx cerebri; Ga: vein of Galen; Ge: genu of the corpus callosum; Is: isthmus of the corpus callosum; pca: pericallosal artery; Sp: splenium of the corpus callosum.)

For manipulations in the deep surgical field, the use of surgical instruments designed for endoscopic use, such as single shaft bipolar coagulators and scissors, and longer-tipped aspiration devices, are mandatory. However, for meticulous procedures such as interhemispheric fissure dissection, manipulations under microscopy are more advantageous. For now, advantages beyond a shorter skin incision and smaller craniotomy have not been reported. In aCC and tCC, we adopt combined use of the microscope and endoscope, i.e., using the microscope to dissect the interhemispheric fissure, and the endoscope to transect the corpus callosum. In pCC, we use the endoscope alone. Intraoperative photographs from endoscopic aCC (Figure 7A–C) and endoscopic pCC (Figure 7D–F) are shown.

### 4.4. CC without Craniotomy

To reduce the invasiveness with craniotomy, stereotactic laser CC [15,16] and radiosurgical CC [17] have recently been developed. These techniques reportedly provide favorable seizure outcomes with low complication rates, comparable to the above-mentioned conventional CC with craniotomy. However, for stereotactic laser CC, multiple trajectories are necessary, and it might increase the risk of damaging anterior cerebral arteries. Radiosurgical CC needs neither craniotomy nor skin incision, but surgeons should be concerned about the acute and late complications following irradiation. To establish these methods as viable alternatives to microsurgical CC, methodological standardization and larger studies are needed.

## 5. Surgical Complications

### 5.1. Hydrocephalus and Subdural Fluid Collection

Especially in cases of brain atrophy, subdural fluid collection might occur after CC. In most cases, this is asymptomatic. Hydrocephalus can be caused by opening the lateral ventricle during callosal section. Entering the CSP without opening the lateral ventricle lowers the risk of postoperative subdural fluid collection and hydrocephalus. If part of the lateral ventricle is opened accidentally, the ventricular wall should be covered with an absorbable gelatin sponge soaked with fibrin glue.

### 5.2. Hemorrhage and Infarction

Subdural hematoma can be caused by damaging bridging veins leading from the brain surface to the SSS. Intraparenchymal hemorrhage can be caused by excessive brain retraction. Infarction in the territory of the anterior cerebral artery can result from damage to the pericallosal or callosomarginal arteries.

In all microsurgical steps, gentle brain retraction, repeated replacement of the spatula, and covering arteries with rayon patties are important to avoid damage to bridging veins, brain parenchyma, and arteries.

## 6. Conclusions

CC can be performed using basic neurosurgical techniques. Considering CC as one of the options in epilepsy surgery to palliate seizures, the procedure is only acceptable under a low prevalence of complications. Surgeons should thus make the maximum effort to minimize the complication rate.

## Data Availability

This article does not include any data which needs availability statement.
